# Supplementary material for: A Bayesian phase 2 model based adaptive design to optimise antivenom dosing: Application to a dose-finding trial for a novel Russell’s viper antivenom in Myanmar
Source: PLoS Negl Trop Dis. 2020 Nov 16;14(11):e0008109. doi: 10.1371/journal.pntd.0008109 (PMC7704047; doi:10.1371/journal.pntd.0008109)
Supplement: S1 Table — (PDF) [file pntd.0008109.s001.pdf]

**S1 Table List of studies selected from literature review**. AR: adverse reaction; AE: adverse event; SAE: serious adverse event; NR: not reported; NT: not tested. NA: not available yet; E: established; N: new. * Not yet published.

| Country (Year) [Ref] | Antivenom | Species | Study design | Status | Published use of pre-clinical data? | Power calculation | Design (N) | N | Efficacy | Toxicity |
|---|---|---|---|---|---|---|---|---|---|---|
| Brazil (1995)[1] | Butantan Bothrops polyvalent antivenom | *Bothrops spp.* | Phase 3, prospective, randomised, double blind | E | Yes (5.0mg/ml) | No | 20mL (88), 40mL (82) | 162 | WBCT | NR |
| India (2004)[2] | Serum Institute of India polyvalent | NT (*Echis carinatus*) | Phase 3, prospective, non-randomised comparative clinical study | E | NR | No | Loading doses 30mL (30), 70mL bolus (30), 100mL bolus (30) | 90 | Clotting time | NS |
| India (1985)[3] | Haffkine polyvalent | NT (*Daboia russelli, Echi carinatus*) | Phase 3, prospective, non-randomised comparative | E | NR | No | Loading dose 40mL (26), 20mL (27) | 53 | Clotting time | NR |
| Nepal (2017)[4] | VINS Indian polyvalent antivenom | *Naja naja* & *Bungarus caeruleus* | Phase 3, prospective, randomised double blind | E | Yes (0.704mg/ml (*B. caeruleus*)) | Yes | Loading doses 20mL (78) and 100mL (79) | 157 | Composite | SAE or AE |
| India (2004)[5] | Serum Institute of India polyvalent | NT | Phase 3, prospective, randomised double blind | E | NR | No | 120mL (50), 60mL (50) | 100 | Clotting time | NR |
| Nigeria (2010) [6] | EchiTAb G, EchiTAb - Plus-ICP, EgyVac | *Echis ocellatus* | Phase 1, prospective, 3+3 | N & E | Yes (EchiTAbG ED50 - 8$\mu$L/mouse and EchiTAb plus 26 $\mu$L/mouse) | N/A | EchiTAb G 10mL (6), EchiTAb - Plus-ICP 30mL (6) and EgyVac 50mL (6) 60mL (6) | 24 | 20WBCT | AR |
| Sri Lanka (1999)[7] | PolongaTab | *Daboia russelli* | Phase 1/2 prospective, non-randomised comparative | N | Yes (1.5mg/mouse) | N/A | 2g (17), 3g (14), 4g (2) | 33 | 20WBCT | NR |
| Nigeria (1997)[8] | EchiTab | *Echis ocellatus* | Phase 1/2, prospective, non-randomised comparative | N | NR | N/A | 20mL (7), 10mL (22) | 29 | 20WBCT | AR |
| Iran (2013)[9] | Razi Vaccine and serum research Polyvalent Antivenom | *Echis carinatus* (90%) | Phase 4, Retrospective cohort | E | NR | No | 40-60mL (78) 50-100mL (76) | 154 | PT or fibrinogen | AR |
| Australia (2004)[10] | CSL Brown snake antivenom | *Pseudonaja spp.* | Phase 4, Retrospective observational | E | NR | No | Dose range between 1-23 Vials | 35 | Fibrinogen | NR |
| Brazil (2003)[11] | Antilonomic serum | *Lonomia obliqua* | Phase 1/2, prospective, randomised, double blind | N | Yes (0.35mg/ml venom) | Yes | 10.5mg (22) and 17.5mg (22) | 44 | Clotting time | Early AR |
| Brazil*[12] | Vital Brazil Institute Apillic antivenom | *Apis mellifera* | Phase 1/2, prospective, non-randomised comparative clinical study | N | Yes (1.25mg/ml venom) | N/A | 0, 20mL, 60mL and 100mL | NR | composite | AE |
| Tunisia (1999)[13] | PIT bivalent antivenom | *Androctonus australis garzonii* & *Buthus occitanus tunetanus* | Phase 4, Retrospective observational | E | Yes (expressed as ratio of LD50 - 10-20LD50/ml) | No | Multiple dosing IV and 4 | 147 | Composite | NR |
| India (2017)[14] | Serum Institute of India and VINLIFE bioproducts | *Daboia russelli and Echis oscellatus* | Phase 3, prospective, non-randomised clinical comparative study | E | NR | No | Loading 100mL (19), Loading 20mL (21) | 40 | Clotting time | NR |
| Myanmar (2001)[15] | Myanmar Pharmaceutical Industry Viper antivenom | *Daboia siamensis* | Phase 3 prospective randomised double blind | E | NR | No | 40mL (23), 80mL (22) | 45 | 20WBCT | NR |
| Iran (2016)[16] | Razi Vaccine and serum research Polyvalent Antivenom | NT (*Echis carinatus*) | Phase 4, Retrospective observational | E | NR | No | WHO (19), Goldfrank's (28), Haddad (5) | 52 | composite | serum sickness |

# References


[1] M. T. Jorge, J. L.C. Cardoso, S. C.B. Castro, L. Ribeiro, F. O.S. Franca, M. E.Sbrogio De Almeida, A. S. Kamiguti, I. S. Santo-Martins, M. L. Santoro, J. E.C. Mancau, D. A. Warrell, and R. D.G. Theakston. A randomized 'blinded' comparison of two doses of antivenom in the treatment of Bothrops envenoming in S5o Paulo, Brazil. *Transactions of the Royal Society of Tropical Medicine and Hygiene*, 89(1):111–114, 1995.

[2] J. Srimannarayana, T. K. Dutta, A. Sahai, and S. Badrinath. Rational use of anti-snake venom (ASV): Trial of various regimens in hemotoxic snake envenomation. *Journal of Association of Physicians of India*, 52:788–793, 2004.

[3] J. Jacob and Paulose P. Thomas. Randomised trial of antivenom in snake envenomation with prolonged clotting time. *British Medical Journal*, 291(6489):177–178, 1985.

[4] Emilie Alirol, Sanjib Kumar Sharma, Anup Ghimire, Antoine Poncet, Christophe Combescure, Chabilal Thapa, Vijaya Prasad Paudel, Kalidas Adhikary, Walter Robert Taylor, David Warrell, et al. Dose of antivenom for the treatment of snakebite with neurotoxic envenoming: Evidence from a randomised controlled trial in nepal. *PLoS Neglected Tropical Diseases*, 11(5):e0005612, 2017.

[5] V. Paul, S. Pratibha, K. A. Prahlad, Jerry Earali, S. Francis, and Francy Lewis. High-dose anti-snake venom versus low-dose anti-snake venom in the treatment of poisonous snake bites - A critical study. *Journal of Association of Physicians of India*, 52(JAN):14–17, 2004.

[6] SB Abubakar, IS Abubakar, AG Habib, A Nasidi, N Durfa, PO Yusuf, S Larnyang, John Garnvwa, E Sokomba, L Salako, et al. Pre-clinical and preliminary dose-finding and safety studies to identify candidate antivenoms for treatment of envenoming by saw-scaled or carpet vipers (*Echis ocellatus*) in northern nigeria. *Toxicon*, 55(4):719–723, 2010.

[7] C. A. Ariaratnam, W. P. Meyer, G. Perera, M. Eddleston, S. A.M. Kuleratne, W. Attapattu, R. Sheriff, A. M. Richards, R. D.G. Theakston, and D. A. Warrell. A new monospecific ovine Fab fragment antivenom for treatment of envenoming by the Sri Lankan Russell's viper (Daboia russelii russelii): A preliminary dose-finding and pharmacokinetic study. *American Journal of Tropical Medicine and Hygiene*, 61(2):259–265, 1999.

[8] W P Meyer, A G Habib, A A Onayade, A Yakubu, D C Smith, A Nasidi, I J Daudu, D A Warrell, R D G Theakston, Epidemiology Unit, Bauchi State, United Kingdom, Social Services, and Bauchi State. First clinical experiences with a new ovine FAB Echis ocellatus snake bite antivenom in Nigeria: Randomized comparative trial with Insititute Pasteur Serum (IPSER) Africa Antivenom. *The American Journal of Tropical Medicine and Hygiene*, 56(3):291–300, 1997.

[9] Ali Hassan Rahmani, Amir Jalali, Mohammad Hassan Alemzadeh-Ansari, Mina Tafazoli, and Fakher Rahimc. Dosage comparison of snake anti-venom coagulopathy. *Iranian Journal of Pharmaceutical Research*, 13(1):283–289, 2014.

[10] Justin M Yeung, Mark Little, Lindsay M Murray, George A Jelinek, and Frank F S Daly. Antivenom dosing in 35 patients with severe brown snake (Pseudonaja) envenoming in Western Australia over 10 years. *Medical Journal of Australia*, 181(11):703–705, 2004.

[11] Jairo José Caovilla and Elvino José Guardão Barros. Efficacy of two different doses of antilonomic serum in the resolution of hemorrhagic syndrome resulting from envenoming by Lonomia obliqua caterpillars: a randomized controlled trial. *Toxicon*, 43(7):811–818, 2004.

[12] Alexandre Naime Barbosa, Leslie Boyer, Jean Philippe Chippaux, Natalia Bronzatto Medolago, Carlos Antonio Caramori, Ariane Gomes Paixão, João Paulo Vasconcelos Poli, Mônica Bannwart Mendes, Lucilene Delazari dos Santos, Rui Seabra Ferreira, and Benedito Barraviera. A clinical trial protocol to treat massive Africanized honeybee (Apis mellifera) attack with a new apilic antivenom. *Journal of Venomous Animals and Toxins Including Tropical Diseases*, 23(1):1–10, 2017.

[13] Mohamed Naceur Krifi, Fethi Amri, Habib Kharrat, and Mohamed El Ayeb. Evaluation of antivenom therapy in children severely envenomed by Androctonus australis garzonii (Aag) and Buthus occitanus tunetanus (Bot) scorpions. *Toxicon*, 37(11):1627–1634, 1999.

[14] Imanto M. Joseph, Cijoy K. Kuriakose, Anand Vimal Dev, and George A. Philip. Low dose versus high dose anti-snake venom therapy in the treatment of haematotoxic snake bite in South India. *Tropical Doctor*, 47(4):300–304, 2017.

[15] WHO/Regional Office for South-East Asia. Management of Snakebite and Research: Report and Working Papers of a Seminar Yangon, Myanmar, 11-12 December 2001. Technical report, World Health Organization, New Delhi, 2001.

[16] Afshin Mohammad Alizadeh, Hossein Hassanian-Moghaddam, Nasim Zamani, Mitra Rahimi, Mohammad Mashayekhian, Behrooz Hashemi Domeneh, Peyman Erfantalab, and Ali Ostadi. The Protocol of Choice for Treatment of Snake Bite. *Advances in Medicine*, 2016:1–5, 2016.
