# Supplementary material for: A Bayesian phase 2 model based adaptive design to optimise antivenom dosing: Application to a dose-finding trial for a novel Russell’s viper antivenom in Myanmar
Source: PLoS Negl Trop Dis. 2020 Nov 16;14(11):e0008109. doi: 10.1371/journal.pntd.0008109 (PMC7704047; doi:10.1371/journal.pntd.0008109)
Supplement: S1 Text — (PDF) [file pntd.0008109.s002.pdf]

S1 Text Bayesian adaptive design details

Adaptively determining the optimal dose for patients randomised to the adaptive arm requires parametric assumptions about the dose-response relationship for both toxicity and efficacy. For the toxicity dose-response model we choose the logistic (sigmoid) model, with the dose v (in mL, 10 mL is equal to one vial) on the logarithmic scale (base 2 for visual simplicity). For the efficacy dose-response model we choose the probit regression as the parameters values are directly interpretable (see main text of Methods).

After the burn-in period, for each new cohort of patients, we estimate the posterior distribution over the model parameters (the two parameters of the logistic toxicity model and the two parameters of the probit efficacy model). In the code provided, the Bayesian update is done using a Monte Carlo method implemented in stan [1]. Our implementation uses 2000 iterations with point estimates used to assign doses in the previous cohort as the starting point for the chain in the next cohort.

We then use the mean posterior parameters to estimate the MTD and TED. Patients in new cohort who are randomised to the adaptive arm are then administered the lower of the estimates MTD and TED (see R function *estimate_posterior_params* in the accompanying code).

The logistic regression for the toxicity dose-response model has two independent parameters: an intercept term which we denote α_{tox}, and a slope term for the $\log_2$ dose which we denote β_{tox}. Under the logistic function parameterisation of the toxicity dose-response model, it is possible to interpret the parameters of the model as follows. The intercept term α_{tox} corresponds to the expected toxicity after administration of one unit of antivenom. The slope term is the log-odds ratio for the toxicity outcome for a doubling of the dose.

At each step of the model update, it is possible to either use all the available accrued data or only the data from the adaptive arm. Under model mis-specification, whereby the models of the dose-response relationships are not in agreement with the true, unknown dose-response relationships, it may in fact be sub-optimal to use all the data. The reason is that the model specification (for example, a single parameter model) may be a good local approximation of the true underlying dose-response, but may be a poor overall approximation. If this were the case, then only using data from doses close to the target dose will provide a better approximation than using all the doses including doses substantially below the target dose. We do not believe that this is a concern in the setup here as we use a mechanistically derived and flexible two parameter dose-response model for the efficacy. Nor is this a concern for the toxicity model as the adaptively assigned doses will, in expectation be less than the MTT which is usually low (less than 10%).

References

[1] Bob Carpenter, Andrew Gelman, Matthew D Hoffman, Daniel Lee, Ben Goodrich, Michael Betancourt, Marcus Brubaker, Jiqiang Guo, Peter Li, and Allen Riddell. Stan: A probabilistic programming language. *Journal of statistical software*, 76(1), 2017.
