# Supplementary material for: A Bayesian phase 2 model based adaptive design to optimise antivenom dosing: Application to a dose-finding trial for a novel Russell’s viper antivenom in Myanmar
Source: PLoS Negl Trop Dis. 2020 Nov 16;14(11):e0008109. doi: 10.1371/journal.pntd.0008109 (PMC7704047; doi:10.1371/journal.pntd.0008109)
Supplement: S2 Text — (PDF) [file pntd.0008109.s003.pdf]

S2 Text '3+3' (cumulative cohort) adaptive design rules

We use the following rules for dose escalation and de-escalation:

1. If fewer than 20 patients have received dose v:
   - If $\hat{\theta}_v^{\text{tox}} \geq 1/3$, decrease dose by δ_v
   - If $0 < \hat{\theta}_v^{\text{tox}} < 1/3$, stay at dose v
   - If $\hat{\theta}_v^{\text{tox}} = 0$, then:
     - If $\hat{\theta}_v^{\text{eff}} = 1$ stay at dose v
     - If $\hat{\theta}_v^{\text{eff}} < 1$ increase the dose by δ_v
2. If 20 or more patients have received dose v:
   - If $\hat{\theta}_v^{\text{tox}} > \text{MTT}$, decrease dose by δ_v.
   - If $0 < \hat{\theta}_v^{\text{tox}} \leq \text{MTT}$, then:
     - If $\hat{\theta}_v^{\text{eff}} \geq \text{TEL} + \epsilon$, decrease dose by δ_v
     - If $\hat{\theta}_v^{\text{eff}} \leq \text{TEL} - \epsilon$, increase dose by δ_v
     - Otherwise, stay at dose v

The free parameter ϵ gives the tolerance level around the target efficacious dose. In the simulation studies, we set it equal to 1% point difference. The differentiation based on 20 patients is due to the choice of MTT and TEL which are 5% and 95%, respectively. Only after giving a dose v to 20 patients or more is it possible for the frequentist estimate to discriminate whether $0 < \hat{\theta}_v^{\text{tox}} \leq \text{MTT}$. This will reduce stochasticity during the recruitment of the first 20 patients.
