# Supplementary material for: A Bayesian phase 2 model based adaptive design to optimise antivenom dosing: Application to a dose-finding trial for a novel Russell’s viper antivenom in Myanmar
Source: PLoS Negl Trop Dis. 2020 Nov 16;14(11):e0008109. doi: 10.1371/journal.pntd.0008109 (PMC7704047; doi:10.1371/journal.pntd.0008109)
Supplement: Attachment — Submitted filename: antivenom dose finding methodology Reply_Reviewers_v2.docx [file pntd.0008109.s004.docx]

**Editorial Summary**

It was difficult to collect reviews amid the pandemic, but at last we succeeded in obtaining four expert reviews that, in our opinion, adequately cover the interdisciplinary breadth of the work presented.
As all reviewers agree regarding the potential importance of this work beyond the Myanmar case study with Daboia siamensis/antivenom, we request a major revision that appropriately addresses all suggestions and queries by these reviewers, and adequately honours any comparable or similar published approaches. This should also include an expanded, full and transparent discussion of the (actual, perceived, potential) limitations of this case study. Inclusion criteria and choice of endpoints are issues that were mentioned by reviewers, and these should consequently be addressed in greater detail. More than one reviewer got stuck at the 20WBCT "controversy" (if there is any) and restoration of blood coagulability after 6 hr (which might not be a predictor of either survival or no need for dialysis or amputation, depending on the particular antivenom used, and evidence to be shown), so more room should be allocated to explaining and discussing this.

We thank the editor for these extensive reviews of our work and for giving us the opportunity to resubmit a revised manuscript. We have re-structured the Introduction (to demonstrate more clearly the general applicability of the work), the Methods (both shortened and re-organised to show how the adaptive method works), and the Discussion (we have added sub-headings to clearly demarcate the different aspects, in particular the limitations of the design and the choice of endpoints). The Discussion highlights that the idea in itself is not novel, but that the main contribution of this work is to provide an easy to re-use resource for other researchers optimizing antivenom regimens. The introduction now notes why the particular endpoints (efficacy and toxicity) were chosen, and explicitly states that these can (should) be changed for different contexts.

We note that one extra author (Dr Min Zaw Oo) has been added to the manuscript. He has helped us make alterations to the Myanmar trial protocol and the design so that it is acceptable for the local ethics committee.

We hope this revised submission is suitable for publication in PLoS NTDS.

**Methods**

Reviewer #1: This is a well written research article. Even though I am not an expert in modelling, I can understand the logic of developing the model. Objectives are clearly presented. The study design is appropriate and the statistical methods are appropriate. There are no ethical concerns in this paper.

We thank the reviewer for these comments and for taking the time to review the manuscript.

Reviewer #2: The authors developed a Bayesian phase 2 model for characterizing the optimal initial antivenom doses, considering toxicity and efficacy endpoints. The rationale of the designed model is understandable. Exclusive and inclusive criteria are considered, despite the chosen resolution of envenoming at 6 hours as endpoint ids not sufficient. The logistic function parameterization of the dose-response model and scenarii are interesting.

We thank the reviewer for these comments. We have added in the Introduction (lines 75-79) and in the Discussion reasons why these endpoints have been chosen and the limitations of these endpoints (lines 402-408).

In the Introduction we have added:

*“Envenoming from different snakes will require different definitions of efficacy and toxicity. In the context of Russell's viper envenoming in Myanmar, the choice of these two binary clinical end-points was pragmatic due to their clinical significance, resource availability and replication of current clinical practice.”*

In the Discussion we now note:

*“The design is dependent on consistent and clinically relevant efficacy and toxicity endpoints. Clinically relevant efficacy endpoints for envenoming resulting in coagulopathy include the 20WBCT (applicable to Myanmar where other clotting assays are not available) and a clinically significant elevation in prothrombin time or INR. The objective clinical endpoint used by Alirol et al. in neurotoxic envenoming could be adapted as an efficacy endpoint in an adaptive design, i.e. the avoidance of ventilation from a starting position of pre-defined neurological impairment [27].”*

It is important to note that the methodology is itself agnostic to the endpoint (the only requirement is that the endpoint is binary). Indeed, the adaptive design can only be as good as the endpoint (this is noted in the Discussion).

Reviewer #3: The objectives of the study are clearly articulated with a clear testable hypothesis stated, the study design is appropriate to address the stated objectives, the population are clearly described and appropriate for the hypothesis being tested and the sample size is sufficient to ensure adequate power to address the hypothesis being tested. Statistical analysis are correct, ethical and regulatory requirements are met.

We thank the reviewer for these comments and for taking the time to look over the manuscript.

Reviewer #4: The description of the trial design needs more details. the models for efficacy and toxicity are both just GLMs with a probit and logit link respectively. But how are these used? That's not at all clear. presumably several doses are used at the start, but then how are new doses chosen? We need to see more details.

We agree upon re-reading the manuscript that there was not enough care to fully explain how the adaptive algorithm works. We have re-written and re-structured the Methods section. The first section of the Methods now explains the adaptive algorithm (without any maths) and the appendix gives exact parametric forms for the models and priors. We have made it clearer that the models are simply generalized linear regression models with probit and logit link functions.

Lines 149-153 (link functions):

*The model based adaptive design necessitates determining parametric dose-response relationships for both the dose-related toxicity and the dose-related efficacy. We model both the efficacy and toxicity outcomes as Bernoulli random variables with dose-dependent parameters estimated under a generalised linear model. For the efficacy outcome we use the probit link function, and for the toxicity outcome we use the logistic link function.*

Lines 102-120 (how the model updates happen and how the dose is assigned in the adaptive arm):

*Patients are enrolled in successive cohorts of a pre-specified size N_cohort_≥1The choice of the value of N_cohort_ is pragmatic as it determines how often it is necessary to update the model. Randomisation is performed at the individual level. We assume that the toxicity and efficacy outcomes for all previously enrolled cohorts of patients are known when a new cohort of patients is enrolled. At the start of the trial there is a ``burn-in'' period (a pre-specified number of patients). During this burn-in period, patients randomised to the adaptive arm are given the starting dose for the adaptive arm, which is the optimal dose under the prior distribution over the model parameters. After burn-in, patients randomised to the adaptive arm are given the posterior predicted optimal dose under the model (the distribution over model parameters is updated using all accrued data). If the model predicted dose is more than any previously trialled dose plus the maximum dose increment δ_v_, then patients are given the maximum allowed dose (the greatest previously assigned dose plus δ_v_).*

**Results**

Reviewer #1: yes. It may be useful for the common reader if the presentation of results can be simplified to make better understanding of the results. Figures are clear and of very good quality.

We have tried to simplify the text in the Results. See marked-up manuscript pdf.

Reviewer #2: Results are described in detail and correlations are depicted well. In the discussion part, some additional details are given to the assessed data.

We thank the reviewer for these comments.

Reviewer #3: The presented analysis match the analysis plan and the results are clearly and completely presented. Also the figures and their quality are sufficiently clear.

Thank you for these comments.

Reviewer #4: The presentation of the results is OK, but I wonder if it could be done more succinctly, by defining a (possibly artificial, i.e. purely for the presentation of the results) stopping rule and presenting how long it takes the methods to reach the rule under the different scenarios. This could then be presented in a single plot, which would be easier to interpret.

Figure 1 shows a summary of the stochastic performance of the model based design versus the rule based design under the 7 scenarios considered. We do appreciate that it’s a bit difficult to comprehend due to the large number of scenarios considered but it is difficult to make it more succinct than shown in that Figure given the number of simulation scenarios considered.

**Conclusions**

Reviewer #1: The limitation of the study is clearly the use of the WBCT20 as a surrogate. The authors have addressed this as a limitation and is acceptable.

Thank you for this comment.

Reviewer #2: The limitation of the analysis to the chosen resolution of envenoming at 6 hours is reported and future development is addressed. Dose-finding trials would benefit from the developed model based on adaptive designs.

Thank you for this comment.

Reviewer #3: The authors show clearly the relevance of their results especially on the management of snake envenomation which is a public health problem in many countries. However, in this paper, authors did not well discuss the limitations to the approach. The conclusion lacks precisions.

We have modified the structure of the Discussion so that there is now a sub-heading “Limitations of the Bayesian model based adaptive design”. This regroups elements that were in the previous submission but hopefully is easier to read.

Reviewer #4: I think the conclusions are OK, although it seems odd to end the discussion with a comment about the 20WBCT criterion. That's not what this manuscript is about, so I don't see that it's necessary.

We have re-organised the Discussion and added sub-headings for clarity. The last section is now “Future work”. This uses the 20WBCT to illustrate why better endpoints are needed to design phase 2 studies. We hope this makes more sense.

**Editorial and Data Presentation Modifications**

Reviewer #1: Since the readership of this article is highly likely to be clinicians, it would be appropriate to simplify and shorten the methods section if possible.

We have shortened the Methods section and moved the more technical part of it (e.g. the mathematical description of the rule based design) to a supplementary appendix.

Reviewer #2: The manuscript submitted by Watson et al., entitled "A Bayesian phase 2 model based adaptive design to optimize antivenom dosing: application to a dose-finding trial for a novel Russell's viper antivenom in Myanmar" is written in basic language and is well understandable. The study is quite relevance: Antivenom's administration is a critical step in healthcare management of SBE, one of the important NTDs. For safety administration, small and effective dose is required and has to be carefully assessed. Simulation approach is useful to better control adverse effects.
The study is well performed and assessed data covers a broad spectrum and includes necessary controls.

Minor comment:
- In the Introduction section it will be useful to describe all the pharmacodynamic markers that are important to be considered, independently of the conducted study.

We have expanded the introduction to highlight the importance of pre-clinical work and of pharmacokinetic and pharmacodynamic assessment in dose-finding methodology. We also now mention pre-clinical assessment in the author summary (“*Dosing is typically based on pre-clinical testing*”).

Lines 12-14:

*An integral part of the antivenom clinical research pipeline is pre-clinical assessment including the use of animal models. Pre-clinical assessment includes characterising the neutralisation of venom induced lethality and reversal of specific toxic effects of the venom, and antivenomics [10, 11].*

- It remains unclear whether the introduced model adapted to dosing Russell's viper envenoming could be standardized/globalized to other lyophilized or liquid SBE specific Antivenoms, taking into consideration the process of antivenom's preparation, manufacturing, and batch to batch differences.

This is a very important point. Explicitly taking into account the different sources of variability within the trial is very important. This is in fact an advantage of the model based approach over a rule based approach. By defining a statistical model of efficacy and toxicity, it is possible to explicitly define the sources of variability and adjust for them. The `3+3’ design or indeed any rule based design cannot do this.

We have added in the Methods section (lines 143-147):

*It may be the case that the antivenom used in the trial is from multiple batches. Batch variation can be an important contributor to variability in both toxicity and efficacy. It is straightforward to add a batch variation term in the adaptive models of efficacy and toxicity. This is an advantage of a model based design over a rule based design.*

Reviewer #3: In this paper, authors did not well discuss the limitations to the approach. Indeed, all Bayesian analyses are dependent on the choice of prior distributions, which can be erroneous. In this study, the prior distributions for the dose-efficacy and dose-toxicity response models and the longitudinal model were kept weak so that the posterior distributions would be mostly shaped by the emerging data. In addition, authors could not be certain that each of the seven simulated scenarios was equally likely, so it is possible that their estimation for the average expected sample size, which was not weighted, might not be accurate.

We have made the paragraph in the Discussion concerning the limitations clearer. Adaptive designs usually consider multiple scenarios without weighting of scenarios as this is difficult to do in an objective way. We do not think that the scenarios are equally likely (scenario 4 is the most likely we believe).

Discussion (lines 460-465):

*A limitation specific to the Bayesian design is the need to specify prior distributions over the dose-efficacy and dose-toxicity models. Prior elicitation is difficult, especially with limited numbers of published clinical trials, lack of post marketing data and antivenom batch variability. It is important to note that the priors do not need to be strongly informative but serve to minimise stochasticity at the start of the trial. Specifying an adequate “burn-in” period alleviates the problem of poorly specified priors.*

Minor Comments
1/ One section combing Author Summary and Abstract sections is preferable.

We are following the PLoS NTD formatting which asks for an abstract along with a separate non-technical author summary limited to 150 words.

2/ Authors defined efficacy as the restoration of blood coagulability within six hours, and toxicity as anaphylaxis For Russell’s viper. Can they explain on which basis they chose these parameters.

These endpoints were chosen because they reflect clinical practice in Myanmar. They are imperfect endpoints (especially for efficacy) but are known to correlate with improved outcomes. This has been further elaborated in both the Introduction and Discussion, see reply above and lines 75-79 and 407-416.

3/ Line 382: As a result, most antivenom dosing is based on animal models that are known to extrapolate poorly to humans. Authors should highlight also the importance of animal experimentation as preclinical phase

Thank you for highlighting this important omission. The importance of pre-clinical testing has been added to the Introduction, see above reply to a comment by reviewer 2.

4/ Why authors did not discuss the possible scenario in the discussion part; this weakness is the lack of a deeper discussion.

The limitations paragraph has been made clearer. We have made the limitations into a subsection in the Discussion (see *Limitations of the Bayesian model based adaptive design*).

5/ References should be updated exp : Line 3 page 2 : « Worldwide, there are as many 3 as 2.7 million people affected by SBE resulting in an estimated 81,000 to 138,000 deaths 4 per year »

Thank you for highlighting this error, this has now been corrected.

Reviewer #4: I would suggest the authors re-arrange the manuscript to emphasise the generality of their work. After the introduction, they should present their adaptive design is a generic way, so that it can be accessible to a wider group (basically, anyone wanting to efficiently find an optimum dose when they have to worry about toxicity). After that, present the D. siamensis envenoming context as a justification for the simulation study.

We thank the reviewer for this excellent suggestion. The Methods section has been changed.

I was slightly surprised that this problem hadn't been looked at before, and a bit of searching suggests that there has been work in this area (e.g. DOI: 10.1002/sim.6124, 10.1080/10543400903280613). The seminal work appears to be DOI: 10.2307/2533268, but it doesn't consider a dose response. I think this body of work needs to be mentioned (I suspect there is more work than this - some might have been done with phase I trials).

We apologise for not putting this work better into the context of the very large body of research done in Bayesian adaptive designs. The main purpose of this paper is not to demonstrate an important methodological advance as from a methodological perspective it is rather straightforward, but instead to highlight to the snake-bite envenoming community that adaptive designs could be really useful for dose optimization.

We agree that the framework in itself (just simple regression models for efficacy and toxicity) is not novel at all. We wrote this with clinicians and trialists interested in antivenom dose optimization as the target audience. We have added the following to the first part of the Discussion:

*We note that concurrent consideration of both toxicity and efficacy in phase 1-2 trials is not novel in itself. Although most adaptive dose-finding designs consider a single outcome variable (either toxicity or efficacy), there are important research areas such as oncology, where concurrent consideration of both outcomes is more efficient and possibly safer [33]. Explicitly modelling both the toxicity and efficacy dose-response curves has been previously proposed, in both a frequentist setting [34] and a Bayesian setting [35, 36]. The main contribution of this work is to (i) highlight the utility of model based adaptive designs in the specific context of antivenom dose-finding, (ii) propose dose-response models for both toxicity and efficacy which are appropriate and meaningful in the context of antivenom dose-finding, and (iii) provide open source software that can be re-used for the design of other trials.*

Minor Comments

l80: Is that the first of November, or the 11th of January?

We have changed to a less ambiguous format! We were using the European convention.

l198-208: This is then the same as a GLM with a binomial response and a probit link.

Yes – this has been added for clarity as mentioned above.

l207: \mu + 1.64\sigma for 95% efficacy, I think.

This was phrased as 1.64\sigma as the necessary dose increase from \mu. We have re-phrased for clarity as suggested.

l216-8: What about the slope?

Apologies for this oversight we have added that the slope is the log-odds ratio for toxicity when doubling the dose of antivenom (as dose is on the log_2_ scale).

**Summary and General Comments**

Reviewer #1: Antivenom dosing studies are rare and when new antivenoms are produced, establishing dosing is always challenging. This article provides new insight into this area from which others can learn.

We thank the reviewer for this comment.

The following comments were written in duplicate – these are replied to above

*Reviewer #2: The manuscript submitted by Watson et al., entitled "A Bayesian phase 2 model based adaptive design to optimize antivenom dosing: application to a dose-finding trial for a novel Russell's viper antivenom in Myanmar" is written in basic language and is well understandable.
The study is quite relevance: Antivenom's administration is a critical step in healthcare management of SBE, one of the important NTDs. For safety administration, small and effective dose is required and has to be carefully assessed. Simulation approach is useful to better control adverse effects.
The study is well performed and assessed data covers a broad spectrum and includes necessary controls.

Minor comment:
- In the Introduction section it will be useful to describe all the pharmacodynamic markers that are important to be considered, independently of the conducted study.*

*- It remains unclear whether the introduced model adapted to dosing Russell's viper envenoming could be standardized/globalized to other lyophilized or liquid SBE specific Antivenoms, taking into consideration the process of antivenom's preparation, manufacturing, and batch to batch differences.*

Reviewer #3: The article entitled « A Bayesian phase 2 model based adaptive design to optimise antivenom dosing: application to a dose-finding trial for a novel Russell's viper antivenom in Myanmar » proposes a Bayesian model based adaptive design for clinical trials aiming to determine the optimal dose of BPI antivenom. The main motivation behind this work is the bias of some clinical studies induced by small sample sizes or ethical constraints as well as the non-standardization of the dose selection for treating envenomation to maintain both efficacy and safety of the treatment. The authors use the case of Daboia siamensis (Eastern Russell’s viper) as an illustration for the application of their method. They argued the effectiveness of "Bayesian adaptive design" over the simpler rule-based design. As far as I know, this is the first application of the Bayesian adaptive design in antivenom research. Thanks to this model, it is possible to integrate prior information from previous clinical studies. The authors did this by mining the literature which identifies 16 papers by querying the Medline database followed by filtering. Therefore, the paper is original and brings a new perspective to a topic that represents a challenge in many countries. From the results, it appears that the model-based design performs better than the simpler ‘3+3’ design. The simulations made in this work showed a quicker convergence of the model-based design except for one (scenario 7) were no model was able to converge. The results showed also a contradiction to previous works about the effective size of the cohort to accurately determine the optimal dose which adds to the value of the author's work. However the conclusion as well as the discussion of the results are not so clear.
In conclusion, overall the paper reads well, the methods are described clearly and the results are well presented. For that this work deserves consideration, however, the current version of the manuscript is affected by some limits.

We thank the reviewer for the in-depth review of our paper. We have re-organised the Discussion and added sub-headings for clarity. There is now a section *Limitations of the Bayesian model based adaptive design*. We hope these changes adequately answer all the concerns.

Reviewer #4: I must apologise for the delay in providing my comments, the current situation has hardly been ideal I'm afraid.

I think this is OK, but the method needs to be presented more clearly. It would be impossible to replicate it just from the description.

We thank the reviewer for taking time over this manuscript and we fully understand that delays were inevitable given the current context. We apologize for not adding enough detailed information in the Methods. Reviewer 1 asked that the details in the methods be reduced as the audience is mainly clinical. However, as noted, this work is only useful if other researchers can replicate the simulations. In light of this we have added a supplementary appendix with details of how the adaptive algorithm proceeds. We also note that all the code is publicly available on github. We have added detailed comments to the code and wrapped the simulation study into an RMarkdown document so that others can easily understand and re-run it. From this perspective the results are fully replicable.
